# A case of clozapine-induced stuttering in a HIV-positive individual

**DOI:** 10.4102/sajpsychiatry.v31i0.2462

**Published:** 2025-07-17

**Authors:** Paidamoyo F. Kambuzuma, Belinda Marais

**Affiliations:** 1 Department of Psychiatry, Faculty of Health Sciences, University of the Witwatersrand, Johannesburg, South Africa

**Keywords:** Clozapine, HIV, stuttering, case report, schizophrenia, EPSE

## Abstract

**Introduction:**

Stuttering, a speech disorder marked by disruptions in fluency, can be developmental or acquired. Acquired stuttering often stems from neurological causes, psychological trauma or certain medications, with antipsychotics such as clozapine implicated in several cases. Clozapine, particularly at higher doses, has been associated with dose-dependent stuttering although the precise mechanisms remain uncertain. While clozapine’s effects dopamine pathways and seizure thresholds are proposed mechanisms, movement disorders such as focal dystonia and prior extrapyramidal symptoms (EPSEs) are also implicated.

**Patient presentation:**

A 40-year-old man with HIV, schizophrenia and a history of previous severe dystonic reaction to typical antipsychotics, developed stuttering after initiation of clozapine. His stuttering was accompanied by involuntary oral movements.

**Management and outcome:**

The patient’s psychotic symptoms were found to be treatment-resistant, unresponsive to trials of two antipsychotics, and thus necessitating the initiation of clozapine. Following clozapine administration, the patient developed stuttering, which was unresponsive to benzodiazepine therapy and showed no abnormalities on electroencephalogram (EEG) assessment. Gradual resolution of stuttering was observed upon clozapine discontinuation and a switch to amisulpiride.

**Conclusion:**

Clozapine was identified as the likely cause of stuttering in the presence of additional risk factors such as HIV and a history of EPSEs.

**Contribution:**

This case highlights the importance of recognising clozapine-induced stuttering.

## Introduction

Stuttering is a speech disorder presenting with disruptions in speech fluency, specifically repetitions of words or sounds, prolonged pauses or the production of elongated sounds within words.^1,2,3^ Stuttering is categorised into developmental or acquired forms.^1,3,4^ Developmental stuttering, which constitutes the majority of cases, typically emerges in childhood with a lifetime prevalence of 5%, whereas acquired stuttering can occur at any age either because of neurological causes (neurogenic stuttering), medications (drug-induced stuttering) or psychological trauma (psychogenic stuttering).^1,3,4,5,6^ The underlying pathophysiological mechanisms leading to acquired stuttering are linked to the underlying disorder or medication, with some theories attributing stuttering to dopamine dysregulation, seizure activity, structural and functional changes in the brain and an abnormal auditory feedback.^1,3,4,5,6,7^

Neurogenic stuttering more frequently affects males than females and usually occurs because of stroke, but other causes include traumatic brain injuries, brain lesions, degenerative or autoimmune disorders (e.g. Parkinson’s disease, multiple sclerosis), epilepsy and infections of central nervous system (e.g. meningitis, HIV).^1,4,5,6^ Drug-induced stuttering can be caused by certain medications, such as selective serotonin reuptake inhibitors, lithium and antipsychotics, in particular olanzapine, risperidone and clozapine, with the highest prevalence of cases reported being because of clozapine, followed by olanzapine.^1,4,8,9,10,11,12,13,14^

Clozapine, an atypical antipsychotic used for treatment-resistant schizophrenia, has a well-documented side effect profile including rare effects such as stuttering.^15,16^ Clozapine-induced stuttering specifically may present as a result from the seizure-threshold lowering effect of clozapine, potentially causing focal seizures, which present as stuttering.^4,8,9,16^

A few cases of acquired stuttering in individuals with HIV have been reported in the literature, some of which responded to treatment with zidovudine (AZT).^17,18^ In South Africa, the use of AZT is presently limited to specific circumstances where first- and second-line antiretroviral therapy (ART) regimens are unavailable or contraindicated. The AZT is associated with adverse effects such as neutropenia and anaemia, which preclude its suitability for use in the management of treatment-resistant schizophrenia, where clozapine remains the treatment of choice.^19,20^ To the authors’ knowledge, this appears to be the first report of stuttering occurring in a HIV-positive patient treated with clozapine.

## Ethical considerations

Informed consent could not be obtained from Mr X as he had been discharged and could not be reached to follow up at the time of writing the report. Permission for publication was granted by the specialised psychiatric hospital, and ethics clearance was obtained from the University of the Witwatersrand Human Research Ethics Committee (No. M200381).

## Patient presentation

Mr X, a 40-year-old man, was admitted to a specialised psychiatric hospital with a history of aggressive and disorganised behaviour, hallucinations, bizarre, grandiose and persecutory delusions, occurring on the background of non-adherence for four years. Mr X was diagnosed with schizophrenia 11 years prior and subsequently diagnosed with HIV approximately five years after his index to psychiatry. He had multiple (more than five) admissions to psychiatric facilities over the years, with a previous severe dystonic reaction to typical depot antipsychotics. Mr X had been initiated on ART during a previous admission, six years prior to this presentation. There was a history of poor outpatient adherence to both psychotropics, ART, to his psychiatric and HIV follow-up appointments, with poor interepisodic functioning. On admission to the hospital, Mr X was uncooperative, guarded, had underlying irritability, delusional thought content and poor insight and judgement. He was asuicidal and ahomicidal. Physical examination was unremarkable. He scored 26/30 on the Montreal Cognitive Assessment (lost points for visuospatial and attention). On admission, his CD4 count was 341 cells/mm^3^ and HIV viral load 3680 copies/mL. Other investigations, including a lumbar puncture and computerised tomography of the brain (CTB), showed no abnormalities.

## Management and outcome

Mr X was reinitiated on risperidone and first-line ART- tenofovir 300 mg, emtricitabine 200 mg and efavirenz 600 mg fixed dose combination. His mood settled, but he remained psychotic. Trials of risperidone and olanzapine were unsuccessful, with olanzapine causing drooling, a jaw tremor, abnormal involuntary movements of the mouth and occasional vocal tics. Orphenadrine was added with limited benefit. He was switched to amisulpride, with some improvement in these side effects, but not his psychosis. Clozapine was commenced at a dose of 25 mg administered at night, with subsequent titration in 25 mg increments every three days. Following this initial phase, dosage increases were made more gradually in accordance with clinical response and tolerability, and his psychosis gradually softened, and his insight improved. Stuttering developed at a dose of 300 mg/day (150 mg mane and 150 mg nocte), one month after clozapine initiation and worsened with increasing doses. The maximum clozapine dose achieved was 400 mg/day (150 mg mane and 250 mg nocte). An electroencephalogram (EEG) was performed and found to be normal. No collateral could be obtained to determine a family history or previous history of stuttering. Benzodiazepine (clonazepam) was trialed without benefit. Because of the distress caused by the stuttering, Mr X was weaned off clozapine and subsequently re-initiated on amisulpride at the end of the clozapine taper. There was no deterioration in his condition, and the stuttering subsided. Approximately after 12 months, Mr X was ultimately discharged on the following medication: amisulpride 400 mg twice daily; TLE one tablet at night; propranolol 20 mg twice daily and orphenadrine 50 mg once daily. At the time of discharge, his CD4 had risen to 501, and his HIV viral load was suppressed to lower than detectable limits. He was referred to his local clinic for follow-up.

## Discussion

A retrospective study conducted in Ireland found that clozapine-induced stuttering occurred in 6 out of 654 patients (0.9%), with no significant gender difference.^21^ A case report from Kenya describes a case of clozapine-induced stuttering, notably occurring in the absence of known risk factors.^22^ Clozapine-induced stuttering has been observed to be a dose-dependent side effect, which tends to occur at dosages ranging from 400 mg to 700 mg per day.^8,9^ In the case of Mr X, his stuttering symptoms began at lower doses and did not improve with the addition of a benzodiazepine, and his EEG was normal.

In many reports of clozapine-induced stuttering, it was found that patients often had a history of severe extrapyramidal side effects (EPS) and/or tardive dyskinesia prior to clozapine initiation. It has therefore been suggested that the risk of stuttering may be because of the combination of clozapine and previous EPS, rather than clozapine alone.^14,23^ It has been suggested that stuttering may be a form of focal dystonia of the orofacial muscles, as both stuttering and dystonias present with similar involuntary movements.^7^ Notably, Mr X presented with both a history of previous severe EPS, as well as involuntary movements and tics which co-occurred with his stuttering.

Treatment approaches for stuttering vary. Consultation with speech-language pathologists is recommended; however, conventional stuttering reduction techniques are generally ineffective for drug-induced stuttering. Speech therapy in these cases often remains a process of trial and error.^9,23^ Although some antipsychotics have been implicated as causes for neurogenic stuttering, some (such as haloperidol, risperidone and olanzapine) have been reported as being beneficial in its management. Anticonvulsants, such as sodium valproate, carbamazepine and levetiracetam, are alternative options.^1,4,9,24^ For clozapine-induced stuttering, case reports have described improvements with either clozapine dose reduction or cessation, as was found with Mr X.^13,14^ In the case of Mr X, stuttering developed after clozapine was initiated and resolved with its discontinuation. Mr X’s longstanding history of HIV and previous EPS may have predisposed him to the development of stuttering.

Mr X’s case highlights the need for heightened clinical vigilance for clozapine-induced stuttering, particularly in patients with HIV and a history of EPS. Early recognition is crucial to prevent distress and guide timely management, such as medication adjustment or discontinuation. In addition, it broadens the differential diagnosis for speech disturbances in HIV-positive individuals and contributes valuable insights to the limited literature on side effects of clozapine, specifically in HIV-positive individuals.

## Conclusion

This case report describes the onset of stuttering in a clozapine-treated HIV-positive patient, with a history of previous severe EPS, which resolved with cessation of clozapine and subsequent treatment with amisulpride. Clozapine was deemed to be the cause, or at least the precipitant, for his stuttering, with HIV and the history of previous EPS being possible contributing factors.
